# Inflammasome Gene Polymorphisms (NLRP3 and NLRC4) and Vitamin D Status in Patients with Multiple Sclerosis

**DOI:** 10.3390/ijms27114681

**Published:** 2026-05-22

**Authors:** Concetta Scazzone, Luisa Agnello, Caterina Maria Gambino, Chiara Bellia, Giuseppe Salemi, Anna Masucci, Sabrina Novara, Marcello Ciaccio

**Affiliations:** 1 Department of Biomedicine, Neurosciences and Advanced Diagnostics, Institute of Clinical Biochemistry, Clinical Molecular Medicine, and Clinical Laboratory Medicine, University of Palermo, 90127 Palermo, Italy; concetta.scazzone@unipa.it (C.S.); luisa.agnello@unipa.it (L.A.); caterinamaria.gambino@unipa.it (C.M.G.); chiara.bellia@unipa.it (C.B.); anna.masucci@unipa.it (A.M.); sabrina.novara02@community.unipa.it (S.N.); 2 Department of Laboratory Medicine, University Hospital Paolo Giaccone, 90127 Palermo, Italy; 3 Unit of Neurology, Department of Biomedicine, Neurosciences and Advanced Diagnostics, University of Palermo, 90127 Palermo, Italy; giuseppe.salemi@unipa.it

**Keywords:** multiple sclerosis, inflammasomes, pyrin domain-containing 3 protein, vitamin D, neuroinflammatory diseases

## Abstract

Multiple Sclerosis (MS) is a neuroinflammatory disorder in which genetic and environmental factors contribute to disease onset. Evidence implicates the inflammasome pathway in MS pathophysiology. However, the interaction between inflammasome-related genetic variants and 25-OH-vitamin D_3_ (25(OH)D_3_) levels remains unclear. 105 MS patients and 109 healthy controls were enrolled. Genotyping of *NLRP3* (rs10754558, rs3806265) and *NLRC4* (rs479333) polymorphisms was performed using real-time PCR. Serum 25(OH)D_3_ levels were measured by high-performance liquid chromatography. Clinical severity was assessed using the Expanded Disability Status Scale (EDSS), Multiple Sclerosis Severity Score (MSSS), annualized relapse rate (ARR), and age at onset. MS patients showed significantly lower serum 25(OH)D_3_ levels than controls. Genotype distributions did not differ significantly under an additive model; however, the *NLRP3* rs10754558 GG genotype was more frequent in MS patients under a recessive model and was significantly associated with disease status after adjustment for sex. Subjects carrying the GG genotype also had significantly lower serum 25(OH)D_3_ levels than CC/CG carriers, independently of sex. No significant associations were observed for *NLRP3* rs3806265 or *NLRC4* rs479333, and none of the investigated variants was associated with EDSS, MSSS, ARR, or age at onset. The *NLRP3* rs10754558 polymorphism may be associated with MS susceptibility and reduced circulating vitamin D levels, suggesting a potential link between inflammasome-related genetic variability and immunometabolic regulation in MS.

## 1. Introduction

The Multiple sclerosis (MS) is an immune-mediated disease of the central nervous system characterized by inflammatory demyelination and neuroaxonal damage leading to progressive neurological disability. It represents one of the leading causes of non-traumatic neurological disability in young adults worldwide [1]. The clinical course is highly heterogeneous, ranging from relapsing–remitting to secondary and primary progressive phenotypes, and reflects a complex interplay between genetic susceptibility, environmental factors, and immune dysregulation. Although the mechanisms underlying MS pathogenesis are not fully understood, both adaptive and innate immune responses play key roles in disease onset and progression [2]. Increasing attention has focused on the contribution of innate immune signaling pathways to neuroinflammation.

Among the different inflammasome complexes, NLRP3 (NOD-like receptor family pyrin domain-containing 3) is the most extensively characterized and has been implicated in several autoimmune and neurodegenerative disorders [3,4]. NLRC4 (NOD-like receptor family CARD domain containing 4), classically associated with antibacterial host defense, has more recently been suggested to modulate inflammatory signaling pathways that may also participate in chronic immune-mediated conditions [5].

Genetic variability within inflammasome-related genes has therefore been proposed to influence immune responsiveness and susceptibility to autoimmune diseases. Single-nucleotide polymorphisms (SNPs) in *NLRP3* and *NLRC4* genes may alter their expression, protein function, or downstream cytokine production, thereby shaping the inflammatory milieu. However, evidence regarding the association between these genetic variants and MS risk or clinical phenotype remains limited and sometimes inconsistent, highlighting the need for further investigation [6,7].

In parallel with genetic determinants, environmental and metabolic factors have been extensively explored in MS research. Through its active metabolite, 1,25-dihydroxyvitamin D (1,25-(OH)_2_D), vitamin D exerts pleiotropic immunomodulatory effects via the vitamin D receptor, which is expressed in multiple immune cell populations [8]. This signaling promotes anti-inflammatory pathways, enhances regulatory T-cell function, and suppresses pro-inflammatory Th1 and Th17 responses. Low serum vitamin D levels have been associated with increased MS risk, higher relapse rates, and greater radiological and clinical disease activity [9,10]. Moreover, vitamin D negatively regulates inflammasome activation at both transcriptional and post-translational levels, suggesting a biologically plausible link between vitamin D status and innate immune signaling [11].

Despite growing recognition of both inflammasome pathways and vitamin D deficiency in MS pathophysiology, their potential interaction remains insufficiently explored. Understanding whether polymorphisms in the *NLRP3* and *NLRC4* genes are associated not only with MS susceptibility but also with vitamin D status and clinical severity may provide new insights into the immunometabolic mechanisms underlying disease heterogeneity and aid in the identification of biomarkers for risk stratification and personalized therapeutic strategies.

In this study, we investigated whether *NLRP3* and *NLRC4* genetic variants are associated with multiple sclerosis susceptibility and with serum vitamin D, clinical disease severity, and age at onset.

## 2. Results

The study populations consisted of 105 MS patients and 109 healthy controls. Most MS patients presented with relapsing-remitting MS (84%), 12% with secondary progressive MS, 3% with clinically isolated syndrome, and 1 patient with primary progressive MS. Table 1 shows the main demographic and clinical characteristics of the study population.

No significant deviation from the Hardy–Weinberg equilibrium was observed for *NLRP3* rs10754558, rs3806265, and *NLRC4* rs479333 genetic variants in patients and controls (all *p* > 0.05). Genetic frequencies of *NLRP3* rs10754558, *NLRP3* rs3806265, and *NLRC4* rs479333 were compared between MS and controls with an additive model, finding no statistically significant differences (Table 1). Using a recessive model, the frequency of *NLRP3* rs10754558 GG genotype was higher in MS patients than in controls, although this difference was of borderline statistical significance (*p* = 0.06), suggesting that an association between this genetic variant and the risk of the disease may be present, but this trend requires confirmation. The association between genotype and disease status was further investigated using binary logistic regression. A significant association between the *NLRP3* rs10754558 genotype and MS was detected. Specifically, carriers of the GG genotype had significantly higher odds of being a case compared with non-carriers (OR = 0.40, 95% CI 0.20–0.78, *p* = 0.010), after adjustment for sex (Table 2). *NLRP3* rs3806265 and *NLRC4* rs479333 were not associated with MS in such analysis.

MS patients have lower levels of serum 25(OH)D_3_ than healthy controls (20 [15–26] vs. 39 [(29–49] µg/L; *p* < 0.01). The distribution of serum 25(OH)D_3_ was further evaluated across *NLRP3* and *NLRC4* genotypes. Significantly, subjects with *NLRP3* rs10754558 GG genotype had lower serum 25(OH)D_3_ than CC and CG carriers (24 [18–33] vs. 28 [21–44] µg/L; *p* = 0.03).

When the effects of sex and *NLRP* genotype on serum 25(OH)D_3_ levels were assessed, a significant main effect of genotype was observed (*p* = 0.005), independently of sex. Sex also showed a significant main effect (*p* < 0.001), whereas no genotype-by-sex interaction was detected (*p* = 0.696).

**Figure 1:**
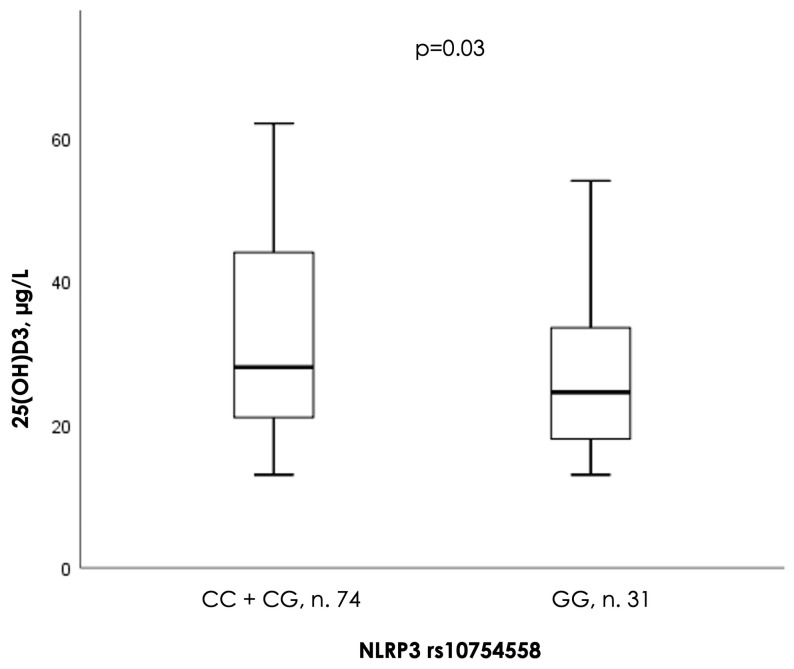
Serum 25(OH)D_3_ level across *NLRP3* rs10754558 genotypes in a recessive model (CC + CG vs. GG).

No significant differences were detected in vitamin D levels across genotypes at *NLRP3* rs3806265 and *NLRC4* rs479333 (Figure 1).

Finally, the association of *NLRP3* rs10754558, *NLRP3* rs3806265, and *NLRC4* rs479333 with disease severity and age at onset was evaluated, finding no significant differences in EDSS, MSSS, ARR, and age at onset across all genotypes (Table 3).

## 3. Discussion

In the present study, we investigated the relationship between selected inflammasome-related genetic variants and circulating vitamin D levels in individuals with MS and healthy controls, focusing on polymorphisms within *NLRP3* and *NLRC4* genes. Although genotype frequencies did not differ significantly between patients and controls, we identified a modest association between the *NLRP3* rs10754558 GG genotype and MS. As expected, MS patients had lower serum 25(OH)D_3_. One of the most important findings of the present study is that *NLRP3* rs10754558 may modulate serum 25(OH)D_3_ levels.

Although a significant gender imbalance was present between MS patients and healthy controls, sex was included as a fixed factor in the general linear model evaluating serum vitamin D levels. This approach allowed us to account for sex-related differences while confirming that the association between *NLRP* genotype and vitamin D levels was independent of sex. These findings suggest that genetic variability within the inflammasome pathway may be associated with differences in vitamin D status and immune regulation in MS.

MS is a chronic immune-mediated disease in which innate immune activation contributes to neuroinflammation and tissue damage. The *NLRP3* inflammasome component has emerged as a key mediator of inflammatory signaling in MS, promoting IL-1β and IL-18 production and microglial activation [3,12,13]. Consistently, genetic variants within inflammasome-related genes, including *NLRP3* and *NLRC4*, have been associated with disease activity and severity, supporting a role for inflammasome pathways in MS pathophysiology [7,14,15].

**Figure 2:**
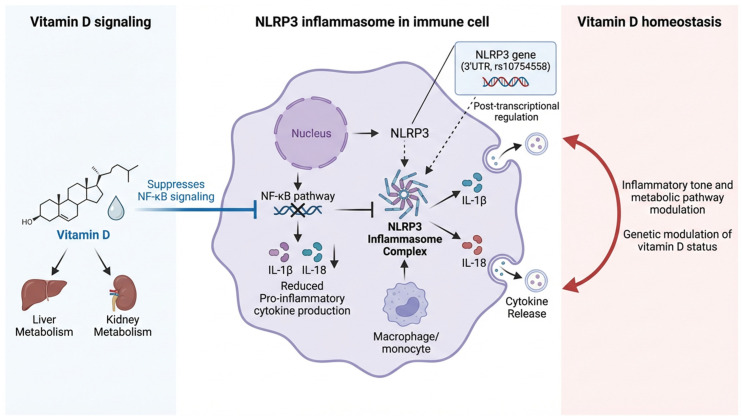
Interplay between vitamin D signaling, NLRP3 inflammasome activation, and vitamin D homeostasis. Vitamin D, following hepatic and renal metabolism, suppresses NF-κB signaling in immune cells, thereby reducing the activation of the NLRP3 inflammasome complex in macrophages/monocytes and limiting the production of pro-inflammatory cytokines (IL-1β and IL-18). Genetic variation in the NLRP3 gene (e.g., rs10754558 in the 3′UTR) may influence post-transcriptional regulation and inflammasome activity. In turn, inflammatory pathways can modulate vitamin D status and metabolic homeostasis, highlighting a bidirectional relationship between immune response and vitamin D regulation.

A functional interaction between vitamin D and inflammasome signaling is biologically plausible. Vitamin D negatively regulates NLRP3 activation by inhibiting NF-κB signaling and reducing downstream pro-inflammatory cytokine production [11,16]. Conversely, genetic variation affecting inflammasome expression may influence inflammatory tone and metabolic pathways linked to vitamin D homeostasis. Notably, rs10754558 is in the 3′UTR of *NLRP3* and affects post-transcriptional regulation of gene expression [17], supporting its functional relevance in inflammatory conditions. The observed association may therefore reflect a bidirectional relationship between inflammasome responsiveness and vitamin D status (Figure 2).

Notably, the *NLRP3* rs10754558 GG genotype was more frequent in MS patients than in controls under a recessive inheritance model. In sex-adjusted analyses, the *NLRP3* rs10754558 GG genotype was associated with a significantly increased risk of disease, whereas the other two loci did not show significant effects. This finding suggests that rs10754558 variant may play a specific role in disease susceptibility, independent of sex. Recent data suggest that recessive models are better than additive models in terms of AUC, discrimination, and precision in genetic association studies [18], providing a more accurate approach for investigating genetic association when two distinct phenotypes are considered, as in case–control study designs. Therefore, *NLRP3* rs10754558, especially when the minor allele is present in double-dose, may be associated with genetic susceptibility to MS. However, these results require confirmation in larger case–control genetic association studies. The absence of differences in the genotype frequencies of *NLRP3* rs3806265 and *NLRC4* rs479333 between patients and controls suggests that these variants are unlikely to represent major susceptibility loci for MS in our population. However, lack of association with disease risk does not exclude functional relevance, as genetic variants may influence intermediate phenotypes without affecting disease prevalence [19]. From a translational perspective, the interaction between vitamin D and inflammasome signaling may have therapeutic implications [20]. Genetic stratification may help identify patient subsets more likely to benefit from immunometabolic pathway-targeted approaches.

No significant associations were observed between the investigated polymorphisms and clinical measures of disease severity (EDSS, MSSS, ARR, or age at onset). This indicates that inflammasome-related variants may affect intermediate immunometabolic traits rather than clinical phenotype, or that their effects require interaction with additional genetic or environmental modifiers.

Vitamin D concentrations are strongly influenced by environmental and behavioral factors (e.g., season, sun exposure, and supplementation), and residual confounding may be present, so this should be considered a limitation of the study. In addition, the relatively modest sample size, which, although appropriate for a preliminary genetic association analysis, may be underpowered to detect small genetic effects, particularly for variants with low minor allele frequency. Furthermore, some of the observed associations reached only borderline statistical significance and should therefore be interpreted with caution. These considerations highlight the need for replication in larger, independent cohorts to confirm our findings. Nevertheless, the observed association between *NLRP3* rs10754558 and vitamin D levels is biologically intriguing and may offer novel insight into the interplay between inflammasome activity and immunometabolic regulation in Multiple Sclerosis (Figure 2).

In conclusion, we identified a genetic association between the *NLRP3* rs10754558 polymorphism and circulating vitamin D levels in MS. Although not directly related to disease severity, this relationship is consistent with a possible role of immunogenetic factors within the immunometabolic network underlying MS.

## 4. Materials and Methods

### 4.1. Study Population

We conducted a retrospective observational case–control study including 105 MS patients and 109 healthy controls. MS patients were recruited at the Unit of Neurology, University Hospital of Palermo “Paolo Giaccone”. Healthy controls were blood donors recruited at the Transfusion Medicine Unit of Villa Sofia-Cervello Hospital, Palermo. At enrolment, demographic and clinical data were recorded, and fasting blood samples were collected.

MS diagnosis was established by an expert neurologist according to the 2024 revised McDonald criteria [21]. The neurological status of patients was assessed using Kurtzke’s Expanded Disability Status Scale (EDSS) [22]. The progression of disability was evaluated using the Multiple Sclerosis Severity Score (MSSS) [23]. The annualized relapse rate (ARR) was calculated during the year preceding enrolment. The study protocol was approved by the Ethics Committee of the University Hospital of Palermo (nr 07/2016) and was performed in accordance with the current revision of the Helsinki Declaration. Informed consent was obtained from all individual participants included in the study.

### 4.2. Molecular Analysis

Peripheral blood samples were collected in EDTA-containing tubes. Genomic DNA was extracted from peripheral blood leukocytes using the chemagic™ magnetic bead–based technology (PerkinElmer, Waltham, MA, USA). Genomic DNA was purified from 200 μL of peripheral whole blood using the QIAamp Blood Mini Kit (Qiagen, Valencia, CA, USA), according to the manufacturer’s instructions. The DNA quality was evaluated by electrophoresis in a 0.8% agarose gel, quantified by absorbance spectrophotometry, and stored at −20 °C for subsequent analysis.

All samples were genotyped by Real-Time allelic discrimination using TaqMan SNP genotyping assays (Applied Biosystems, Foster City, CA, USA). Genotyping of *NLRP3* rs10754558, rs3806265, and *NLRC4* rs479333 polymorphisms was performed using TaqMan SNP Genotyping Assays (Applied Biosystems) on the 7500 Real-Time PCR System (Applied Biosystems, Foster City, CA, USA) (Table 4). Negative controls and duplicate samples were included to ensure genotyping accuracy. Each PCR reaction was carried out in a final volume of 20 μL, containing 25 ng genomic DNA, 5 μL TaqMan Genotyping Master Mix, 0.25 μL TaqMan SNP Genotyping Assay, and nuclease-free water. Thermal cycling conditions were as follows: initial denaturation at 95 °C for 10 min, followed by 40 cycles of denaturation at 95 °C for 15 s and annealing/extension at 60 °C for 1 min, with a final extension at 60 °C for 30 s.

### 4.3. Biochemical Analysis

25(OH)D_3_ levels were measured in serum. Serum samples were obtained from whole blood collected in dry tubes, which were centrifuged serum was stored a −80 °C until analysis. Serum 25(OH)D_3_ was measured by high-performance liquid chromatography (HPLC) using a commercial reagent kit (Chromsystems Instruments & Chemicals GmbH, Gräfelfing, Germany). Serum 25(OH)D_3_ levels were classified as sufficient (>30 µg/L), insufficient (20–30 µg/L), or deficient (<20 µg/L), according to Holick MF et al. [25].

### 4.4. Statistical Analysis

Continuous variables were expressed as mean ± standard deviation (SD) or median and interquartile range (IQR), as appropriate; categorical variables were reported as absolute frequencies and percentages. The normality of data distribution was assessed using the Shapiro–Wilk test. Comparisons between two independent groups were conducted using Student’s *t*-test or the Mann–Whitney test. Differences among more than two groups were evaluated using one-way ANOVA with Bonferroni post hoc correction or the Kruskal–Wallis test for non-normally distributed variables. Categorical variables were compared using the chi-square test or Fisher’s exact test. All genotypes were tested for Hardy–Weinberg equilibrium by using an exact test. Binary logistic regression was performed to evaluate the association between genotype and case–control status, adjusting for sex. Disease status (case/control) was used as the dependent variable, while genotype (coded according to a recessive model) and sex were included as independent variables.

Serum vitamin D levels were analyzed using a general linear model (GLM) univariate analysis with NLRP rs10754558 genotype and sex as fixed factors. The genotype × sex interaction was tested. Homogeneity of variances was assessed using Levene’s test.

A *p* value < 0.05 was considered statistically significant. All statistical tests were performed using SPSS statistical software (version 25, IBM Corp., Armonk, NY, USA).

## Figures and Tables

**Table 1:** Main characteristics of the MS patients and controls. Values are expressed as mean and standard deviation, median and interquartile range, or absolute frequencies and percentages, when appropriate. EDSS: Expanded Disability Status Scale; MSSS: Multiple Sclerosis Severity Score; ARR: annualized relapse rate.

	Multiple Sclerosis	Controls	*p* Value
N	105	109	--
Age, years	39.8	39.9	0.97
Sex, males n (%)	25 (24.8%)	59 (54%)	<0.01
NLRP3 rs10754558, n (%)			0.15
*Additive model*			
CC	30 (28.6%)	34 (31.2%)	
CG	44 (41.9%)	55 (50.5%)	
GG	31 (29.5%)	20 (18.3%)	
*Recessive model*			0.06
CC + CG	74 (70.5%)	89 (81.7%)	
GG	31 (29.5%)	20 (18.3%)	
NLRP3 rs3806265, %			0.63
*Additive model*			
TT	44 (42.7%)	49 (45.0%)	
CT	50 (48.5%)	47 (43.1%)	
CC	9 (8.7%)	13 (11.9%)	
*Recessive model*			0.48
TT + CT	95 (91.3%)	96 (88%)	
CC	10 (8.7%)	13 (11.9%)	
NLRC4 rs479333, %			0.93
*Additive model*			
GG	27 (26.0%)	30 (27.5%)	
GC	56 (53.8%)	59 (54.1%)	
CC	21 (20.2%)	20 (18.3%)	
*Recessive model*			0.73
GG + GC	84 (79.8%)	89 (81.7%)	
CC	21 (20.2%)	20 (18.3%)	
25(OH)D_3_, µg/L	20 (15–26)	39 (29–49)	<0.01
EDSS	2.9 ± 2.1	--	--
MSSS	3.7 ± 2.8	--	--
ARR	1.2 ± 0.9	--	--
Age at onset, years	28 ± 8	--	--

**Table 2:** Association between genetic variants and disease status under a recessive genetic model. Odds ratios (ORs) and 95% confidence intervals (CIs) were estimated using binary logistic regression, with disease status as the dependent variable and genotype as the independent variable, adjusting for sex. In bold statistically significant *p* value.

Genetic Locus	Odds Ratio (OR)	95% CI	*p* Value
*NLRP3* rs10754558 GG	0.40	0.20–0.78	**0.010**
*NLRP3* rs3806265 CC	1.95	0.74–5.12	0.177
*NLRC4* rs479333 CC	1.08	0.52–2.23	0.839

**Table 3:** Distribution of severity scores (EDSS, MSSS, ARR) and age at onset across *NLRP3* and *NLRC4* genotypes. Data are expressed as median (IQR). EDSS: Expanded Disability Status Scale; MSSS: Multiple Sclerosis Severity Score; ARR: annualized relapse rate.

	*NLRP3*, rs10754558				*NLRP3*, rs3806265				*NLRC4*, rs479333			
	CC	CG	GG	*p*	TT	CT	CC	*p*	GG	GC	CC	*p*
EDSS	2 (1.8–4)	3 (1–6)	2 (1–4)	0.40	2 (1–4.4)	2.2 (1.5–5)	3.7 (1.6–6.7)	0.28	3.7 (2–6)	2 (1–4)	2.5 (1–5.2)	0.14
MSSS	2.6 (1–5.2)	4.5 (1.7–7.3)	2.4 (1.6–5)	0.42	2 (1.2–4.7)	4.7 (1.8–6.2)	3.5 (0–6–7.3)	0.14	4.9 (2.2–6)	2.4 (1.1–4.9)	4.9 (0.8–7.5)	0.16
ARR	1 (0–2)	1 (1–2)	1 (1–2)	0.74	1 (0–2)	1 (1–2)	0 (0–1.7)	0.06	1 (1–2)	1 (0–2)	1 (0.5–2)	0.89
Age at onset	29 (24–30)	26 (21–34)	28 (24–32)	0.88	28 (21–32)	28 (23–33)	28 (23–37)	0.47	28 (23–32)	27 (21–32)	28 (25–34)	0.56

**Table 4:** Description of the *NLRP3* and *NLRC4* genetic variants considered in the study.

Gene	Chromosome	SNP	Ancestral Allele	Substitution Allele	Classification	Functional Effect
*NLRP3*	1	rs10754558	C	G	Transversion substitution	Regulates *NLRP3* mRNA stability and downstream inflammasome activation [24]
		rs3806265	C	T	Transition substitution	Influences *NLRP3* expression and inflammasome-driven inflammatory response [14]
*NLRC4*	2	rs479333	C	G	Transversion substitution	Reduces *NLRC4* expression and IL-18 release, attenuating inflammasome activation [7]

## Data Availability

The data presented in this study are available on request from the corresponding author. The data are not publicly available due to privacy.

## Abbreviations

The following abbreviations are used in this manuscript:

MS	Multiple Sclerosis
CNS	Central Nervous System
NLRP3	NOD-like receptor family pyrin domain containing 3
NLRC4	NOD-like receptor family CARD domain containing 4
SNP	Single Nucleotide Polymorphism
DNA	Deoxyribonucleic Acid
PCR	Polymerase Chain Reaction
EDTA	Ethylenediaminetetraacetic Acid
EDSS	Expanded Disability Status Scale
MSSS	Multiple Sclerosis Severity Score
ARR	Annualized relapse rate
HPLC	High-Performace Liquid Chromatography
25(OH)D_3_	25-hydroxyvitamin D3
1,25-(OH)_2_D	1,25-dihydroxyvitamin D
IL	Interleukin
GWAS	Genome-Wide Association Study
AUC	Area Under the Curve
